# Challenges and Barriers Toward Medical Research Among Medical and Dental Students at King Khalid University, Abha, Kingdom of Saudi Arabia

**DOI:** 10.3389/fpubh.2021.706778

**Published:** 2021-08-20

**Authors:** Safar Abadi Alsaleem, Muhammed Alhussain Y. Alkhairi, Mohammed Atiah A. Alzahrani, Mohammed Ibrahim Alwadai, Saad Saleh A. Alqahtani, Yahya Faye Y. Alaseri, Mohammed Awadh M. Alqarni, Safiah Ahmad Assiri, Mohammed Abadi Alsaleem, Syed Esam Mahmood

**Affiliations:** ^1^Department of Family and Community Medicine, College of Medicine, King Khalid University, Abha, Saudi Arabia; ^2^College of Medicine, King Khalid University, Abha, Saudi Arabia

**Keywords:** challenges, barriers, medical, dental, students, research

## Abstract

**Background:** Medical research is a systematic method to obtain new knowledge, reduce diagnosis problems, discover the latest or best treatment for a disease, and also used for disease prevention.

**Objective:** The study aims to assess the challenges and barriers to conduct medical research among medical and dentistry students and determine the reasons that hinder the conduct of the study.

**Methods:** In this cross-sectional study, the research population consisted of medical and dental students affiliated with King Khalid University in Saudi Arabia in 2020. The study followed a descriptive approach based on quantitative analysis. The Statistical Package of the Social Sciences (SPSS) program (V. 16.0, SPSS Inc., Chicago, IL, USA) was used for data analysis.

**Results:** A total of 327 students participated in this study, and the response rate was 100%. Among them, 61.5% were females. In this study majority (63.3%) had a moderate attitude toward conducting medical research and the average was 56.17. The majority (53.8%) had a reasonable behavior toward conducting medical research, and the average was 29.06. Lack of time, skills, funding, facilities, and limited access to medical journals and related databases were the significant barriers found. Most of the students had positive attitudes, except that they are not awarded on their research, do not attend the sessions, and do not speak their thoughts. A significant relationship between demographic characteristics (age, level, and Grade Percentage Average), attitudes & perceived barriers toward research was found (<0.05).

**Conclusion:** The majority of the students demonstrated a positive attitude toward and moderate behavior of research. However, most are not awarded on their research, do not attend the sessions, and do not speak their thoughts. Intensive training and adequate support in research activities of medical and dental students at the undergraduate level may help reduce these challenges and barriers toward research.

## Introduction

Medical research is a systematic method to obtain new knowledge, solve problems such as diagnosis problems, discover the latest or best treatment for such disease, and prevent any disease (1). Knowledge is essential to conduct research which is a barrier in most undergraduate students (2). Research begins with the idea that resulted from a question that needs an answer, considers correlate topics as literature review, set objectives, and chooses appropriate study design to fulfill the objectives. Then, determination of target population, calculating sample size that must be a representative sample, choosing validated and reliable tools that help achieve the goals—finally writing a proposal, getting approval from the ethical committee, collecting and analyzing the data, and writing a proposal research report.

It is essential for medical students to learn and conduct medical searches since the students play a role in the development of nations as future researchers (3). Only 20% of undergraduate student say there is no adequate time to do research during medical school, medical student suffering from lack of training and guidance with research supervisors (4). And many studies said that the early participation in the research will promotes a tendency to keep going research in their later life (5). Even if medical students do not participate to practice clinical research, research knowledge will improve their ability to make evidence based decisions in their clinical life (6) studies have showing that students in developing countries face more barriers in conducting research than students in developed countries (7) some studies show that 43.9% of student had positive attitude toward research (8) and 34.4% was conducting research and only 17.4% of them had published in journals (9) and some studies said that there is no difference in the extent of research participation among students from different high school backgrounds (10). Previous reports have demonstrated awareness of the importance of research in dentistry among dental students (11–13). The majority of Medical students in their undergraduate period are interested in conduct research, but unfortunately, there are some barriers and challenges that prevent them from doing it (5). Limited level of knowledge to conduct research and very low practice are considered barriers. The majority of students are convinced that conducting research is exhausting and stressful (14) and lack access to information sources and lack of expertise in the English language (2). Lack of mentorship during medical school years (15) 46% of Postgraduates believe that a research time should be added to the medical school curriculum (4). These barriers are more in developing countries (16). Prior studies from Saudi Arabia have also reported such barriers (17, 18). Therefore, it was important to find out the challenges and barriers faced by the students in doing research and try to develop recommendations that may contribute to solving the problem. Inadequate skills and knowledge in research methodologies, limited access to information sources, and limited facilities are the main barriers to research in University students. Holding theoretical and practical research methodology courses (2, 19), forming a responsive and helpful research team assistant to support students, providing them with required facilities/equipment, and providing more financial support for students' research activities can help remove barriers to research. The main barriers are lack of time, financial aid, good inter-departmental coordination, and adequate documentation of patient records. Identifying these barriers may help dental authorities to intervene to improve the research-friendly environment (20). Medical students face the main barriers in the research: lack of knowledge, lack of time, lack of guidance, and lack of funding (21, 22). Teaching and learning take place to impart knowledge that teachers possess and make students' learning experience worthwhile and fruitful; based on Bilal et al. (23), Previous exposure to scientific research experiences promotes more positive attitudes toward scientific research (24). Despite the importance and benefits of undergraduate research, attempts of medical schools to encourage undergraduates to take part in formal research training during undergraduate medical education remain unsatisfactory (25). The students demonstrated good research knowledge, but their position was not up to the mark (26). Also, no such research has been conducted before from King Khalid University among medical and dental students. This research fills the literature gaps by reporting the challenges and barriers toward medical research among medical and dentistry students and providing the reasons that hinder the conduct of the research.

The barriers raised point to the need to change search strategies. The study aims to assess challenges & barriers among medical and dental students to conduct medical research, evaluate the challenges and barriers regarding conducting research and to determine the reasons that obstruct conduct research.

## Materials and Methods

### Research Design

This was a 6 month cross-sectional study conducted from July to December 2019. The research population consisted of medical students affiliated with King Khalid University in Saudi Arabia in 2020. The sample size was estimated based on an equation N = (Z_α/2_)^2^ s^2^/d^2^, where s is the standard deviation obtained from previous study or pilot study, and d is the accuracy of estimate or how close to the true mean. Z_α/2_ is a normal deviate for the two-tailed alternative hypothesis at a level of significance. The maximum *P* level was set at 0.5, the confidence level was set at 95%, and the estimated error was 5.37%. The final sample size was determined to be 327. The participants were chosen from among the research students. In addition, a student with experience in research activities such as participating in research projects, presenting papers at national and international conferences, and publishing a paper in English journals was included. Those with no experience in the research were excluded. Sample subjects were selected by stratified relative random sampling. In this regard, each college was taken as a class. Subsequently, the required materials were selected from each layer, in proportion to the size of each layer, using a simple random method. Therefore, a total of 327 subjects were selected. Students were contacted between class lectures or teaching sessions and invited to participate in the survey by study representatives who were also students in the respective medical school at the time. Participation in this study was entirely voluntary, and confidentiality and anonymity were assured. Written consent was obtained from those who agreed to participate.

### Instrument

The data collection Instrument was a paper-based interview triple questionnaire. The first part included demographic information, the second part concerning Barriers experienced by medical students in conducting research, which consisted of 15 items, and the last part concerned Attitudes of the student toward research, which included 14 items. The second and third sections of the questionnaire were scored using a Likert scale ranging from “completely agree” (grade 5) to “completely disagree” (grade 1). Yes (grade 2) and no (grade 1). Moreover, the total score for each item was calculated. A higher score means greater agreement with barriers to research work. The average score was estimated for each domain (personal or organizational). The targeted questionnaire was developed based on the literature review and relevant scientific sources. Both quantitative and qualitative procedures were followed to establish the external validity of the questionnaire. For the qualitative verification of the face, 20 University students were interviewed face to face. The level of difficulty (difficulty understanding phrases), relevance (relevance of data to survey dimensions), and ambiguity (potential misunderstanding or failure to understand the meanings of the data) of the items were investigated. Once corrections were made, the next step was to reduce the statements, ignore repetition, and determine the significance of each statement through the quantitative method of estimating the elements' impact. Handicap was defined as a disadvantage resulting from impairment or disability that limits an individual's social role, e.g., inability to work somewhere due to limited access.

For attitudes, for positive items, strongly agree was scored five, and strongly disagree was scored one. In contrast, for negative items, strongly agree was scored one, and strongly disagree was scored five (higher score indicates better attitude). Regarding perceived barrier, disagree was scored three, score one for agree, and score two for undecided (higher score indicates lesser perceived barrier). The total score was computed by taking the sum for all of these. We categorized attitudes into three levels such as strong (>80% of the maximum possible total score), moderate (60–80% of the maximum possible total score), and poor (<60% of the maximum possible total score).

### Validity and Reliability

To validate the content: a questionnaire was available to five faculties to verify the simplicity of each statement and the relevance and clarity of each statement. To determine reliability: a test method - retest was used. For this purpose, a group of 15 subjects was presented with a questionnaire twice at a 10-day interval, and the resulting data were checked for association.

### Data Analysis

The data analyzed by the Statistical Package for Social Sciences (SPSS) program (V. 16.0, SPSS Inc., Chicago, IL, USA). Descriptive statistics such as mean, standard deviation, frequency, and percentage were calculated. Inferential statistics were also used, including the *t*-test of independent samples, one-way analysis of variance, and Pearson's correlation coefficient. The significance level was set at *P* < 0.05.

## Results

A total of 327 students participated in this study, and the response rate was 100%. Among them, 61.5% are females, 38.5% are males, and the average age of the respondents was 22.52. Most of the respondents were in the 6th year level (27.8%) with an average of 4.49, and the majority was Medical College with a percentage of 78.9% and more Grade Percentage Average (GPA) for students 4.49-4 with a rate of 34.6% with an average of 2.99 (Table 1).

**Table 1:** Demographic characteristics of medical and dental students (*n* = 327).

**Variables**	**Frequency (%)**
**Sex**	
Male	126 (38.5%)
Female	201 (61.5%)
**Age**	
Mean (SD)	22.52 (1.74)
**Level**	
1st year	8 (2.4%)
2nd year	37 (11.3%)
3rd year	55 (16.8%)
4th year	57 (17.4%)
5th year	53 (16.2%)
6th year	91 (27.8%)
Intern	26 (8.0%)
Mean (SD)	4.49 (1.61)
**College**	
Medical College	258 (78.9%)
Dental College	69 (21.1%)
**GPA**	
<2.4	3 (0.9%)
3.9–2.5	105 (32.1%)
4.49–4	113 (34.6%)
4.5 or More	106 (32.4%)
Mean (SD)	2.99 (0.83)

In this study, only 17.1% of the students had few handicaps, while 19.6% had major handicaps. Still, the majority (63.3%) had a moderate attitude, and the average was 56.17, and 9.2% of the students had poor behavior. In comparison, 37% had good behavior, but the majority (53.8%) had a moderate position, and the average was 29.06, which is indicated in Table 2.

**Table 2:** Attitudes, perceived barriers toward research, and previous research experience among medical and dental students.

**Variables**	**Frequency (%)**
**Barriers experienced by medical students in conducting research**	
Poor	56 (17.1%)
Moderate	207 (63.3%)
Good	64 (19.6%)
Mean (SD)	56.17 (7.53%)
**Attitudes of the student toward research**	
Poor	30 (9.2%)
Moderate	176 (53.8%)
Good	121 (37.0%)
Mean (SD)	29.06 (4.74%)

The percentage of answers to the position questions among undergraduates is shown in Table 3, where all items are located between agree and agree strongly barriers except that the students do not find the motivation of faculty members, they find the permanent motivation of them this concerning Barriers while in terms of attitudes.

**Table 3:** Barriers experienced by medical students in conducting research.

**Statements**	**Strongly disagree**	**Disagree**	**Neutral**	**Agree**	**Strongly agree**
	**Frequency (%)**	**Frequency (%)**	**Frequency (%)**	**Frequency (%)**	**Frequency (%)**
Lack of knowledge Lack of knowledge	3 (0.9%)	27 (8.3%)	67 (20.5%)	104 (31.8%)	126 (38.5%)
Lack of time	4 (1.2%)	22 (6.7%)	54 (16.5%)	92 (28.1%)	155 (47.4%)
Inadequate skills in doing research	1 (0.3%)	19 (5.8%)	38 (11.6%)	94 (28.7%)	175 (53.5%)
Inadequate skills in statistics methods	3 (0.9%)	25 (7.6%)	57 (17.4%)	95 (29.1%)	147 (45%)
Lack of finance	11 (3.4%)	48 (14.7%)	88 (26.9%)	74 (22.6%)	106 (32.4%)
Limited database access	6 (1.8%)	20 (6.1%)	75 (22.9%)	103 (31.5%)	123 (37.6%)
Lack of interest	11 (3.4%)	47 (14.4%)	77 (23.5%)	78 (23%)	114 (34.9%)
Difficulty in choose topic	10 (3.1%)	43 (13.1%)	62 (19%)	108 (33.0%)	104 (31.8%)
Getting permission from Ethical committee	4 (1.2%)	22 (6.7%)	146 (44.6%)	66 (20.2%)	89 (27.2%)
Difficulty in writing proposal	2 (0.6%)	19 (5.8%)	130 (39.8%)	87 (26.6%)	89 (27.2%)
Difficulty in collecting data	2 (0.6%)	52 (15.9%)	73 (22.3%)	119 (36.4%)	81 (24.8%)
Difficulty in analysis	2 (0.6%)	38 (11.6%)	92 (28.1%)	113 (34.6%)	82 (25.1%)
Difficulty in writing report	7(2.1%)	37 (11.3%)	106 (32.4%)	99 (30.3%)	78 (23.9%)
Lack of rewarding and/or motivation	8 (2.4%)	26 (8.0%)	78 (23.9%)	76 (23.2%)	139 (42.5%)
Did you find any motivation from faculty in your college to do conduct research	131 (40.1%)	84 (25.7%)	65 (19.9%)	27 (8.3%)	20 (6.1%)

Table 4 shows that most of the behaviors were positive for students, except that they are not honored on their research and not to attend the sessions and do not speak of their thoughts.

**Table 4:** Attitudes of the students toward research.

**Statements**	**No**	**Yes**	**Not inferred**
	**Frequency (%)**	**Frequency (%)**	**Frequency (%)**
Do have an interest in doing research?	57 (17.4%)	270 (82.6%)	0 (0%)
Do you plan to continue working with research after graduation?	71 (21.7%)	256 (78.3%)	0 (0%)
Do you believe in the importance of medical researches?	11 (3.4%)	316 (96.6%)	0 (0%)
Have ever participated in any research?	169 (51.7%)	158 (48.3%)	0 (0%)
Did you publish it?	112 (34.3%)	46 (14.1%)	169 (51.7%)
Did get awarded for your research?	150 (45.9%)	8 (2.4%)	169 (51.7%)
Have you ever attended a course in research?	213 (65.1%)	114 (34.9%)	0 (0%)
Do you have an idea which you think it's good for research?	165 (50.5%)	162 (49.5%)	0 (0%)

Table 5 also measures the students' attitudes, as all items appear between agreeing and strongly agree.

**Table 5:** Attitudes of the students toward research.

**Statements**	**Strongly disagree**	**Disagree**	**Neutral**	**Agree**	**Strongly agree**
	**Frequency (%)**	**Frequency (%)**	**Frequency (%)**	**Frequency (%)**	**Frequency (%)**
What (in your opinion) could help you to do research?	20 (6.1%)	23 (7.0%)	38 (11.6%)	87 (26.6%)	159 (48.6%)
Academic schools or courses in researches	5 (1.5%)	7 (2.1%)	26 (8.0%)	100 (30.6%)	189 (57.8%)
Distribute the student among the college faculty to help them do research	4 (1.2%)	8 (2.4%)	11 (3.4%)	69 (21.1%)	235 (71.9%)

In Table 6, there is no relationship between gender and barriers. At the same time, there is a relationship between sex and attitudes, the majority was for males, and concerning age, there was a relationship between age, barriers, and attitudes. Barriers were more for those aged 25–27 years and attitudes for those aged 23–24 years. Between the relationship between level, barriers, and attitudes, and was in favor of interns. As for the college, there is a relationship between the college and barriers, while there is a relationship between the faculty and attitudes. Most medical colleges and GPA found an association between GPA and barriers and attitudes and barriers were more for <2.4 and attitudes for which they are 4.49–4.

**Table 6:** The relationship between demographic characteristics and attitudes and perceived barriers toward research.

**Variables**	**Barriers**		**Attitudes**	
	**Mean (SD)**	***P***	**Mean (SD)**	***P***
**Sex**				
Male	56.58 (7.55)	0.435	29.74 (4.93)	0.040^*^
Female	55.91 (7.53)		28.63 (4.59)	
**Age**				
19**–**22	54.25 (6.94)	0.000^**^	26.31 (4.10)	0.000^**^
23**–**24	57.64 (7.81)		31.55 (4.06)	
25**–**27	58.33 (7.13)		30.82 (3.48)	
**Level**				
1st year	55.50 (12.12)	0.000^**^	23.88 (2.64)	0.000^**^
2nd year	51.76 (6.42)		24.54 (4.17)	
3rd year	53.35 (6.45)		25.65 (3.45)	
4th year	56.93 (5.97)		28.05 (3.63)	
5th year	57.02 (7.08)		31.06 (4.10)	
6th year	57.44 (7.98)		31.99 (3.79)	
Intern	60.77 (7.66)		32.15 (2.89)	
**College**				
Medical College	56.40 (7.64)	0.276	29.35 (4.69)	0.032^*^
Dental College	55.29 (7.09)		27.97 (4.81)	
**GPA**				
<2.4	59.33 (1.15)	0.004^**^	29.00 (7.21)	0.002^**^
3.9–2.5	56.73 (6.83)		29.83 (4.49)	
4.49–4	57.53 (8.33)		29.68 (4.54)	
4.5 or more	54.07 (6.98)		27.63 (4.88)	

* *significant*,

** *highly significant*.

## Discussion

We conducted the study to assess attitudes and barriers to conducting scientific research among undergraduate medical and dental students at our private medical college. An understanding of scientific methods becomes a critical component of the medical profession. Although not every health professional is inspired to research to gain new knowledge, he must know the principles of scientific research (27). In this study, we found that 63.3% of the students had moderation and 53.8% had a moderate level of attitude, with a mean score of 29.06, which is indicated by previous studies as moderating attitude (28, 29). Students also showed positive attitudes toward science and scientific method in medicine. Like other studies, many of our participants demonstrated fairly high confidence in conducting research activities such as clinical questioning, research, and evaluation of the literature. Still, many of them expressed little confidence in accessing clinical experience from the trainer and using evidence-based processes. If health professionals have recognized the ability to perform research activities, they are more likely to be involved in medical research (30, 31). A recent study conducted in Saudi Arabia shows that the difficulty score decreased significantly with increasing the number of clinical research workshops attended by the researchers (32). Lack of reward and/or motivation was a barrier found in our study. The financial rewards can be regarded as a form of reciprocal appreciative exchange for students' diligence (24). In this study, there is no relationship between gender and barriers. While there is a relationship between sex and attitudes, the majority was for males, and concerning age, there was a relationship between age, barriers, and attitudes, and barriers were more for those aged 25–27 years and attitudes for those aged 23–24 years. Our findings are comparable to prior studies, where the relationship between individual characteristics such as age, gender, year of education, and attitudes toward health research (33) was studied, and it was found that there is no relationship between gender and barriers for college students (34, 35). There was a relationship between age, barriers, and research attitudes for students at the Faculty of Medicine, and the barriers were more for those between the ages of 25 and 27 and attitudes for those between the ages of 23 and 24. There was a relationship between college and research barriers in the current study. For medical students GPA, the relationship between GPA, barriers, positions, and barriers were more than 2.4 and positions that were 4.49–4 in our study. Our findings are comparable to those reported by Amin et al. (36) and Pallamparthy et al. (1). It is essential to drive students to develop positive attitudes toward undergraduate research and resolve any barrier that may hinder medical students from productive engagement in undergraduate research activities (24). Even if the medical student does not wish to pursue a research activity/career, one should be well-prepared to read scientific journals critically and decide on proper clinical decisions based on critical interpretation of scientific evidence (37). Enhancing the research funding, job salaries, and productivity incentives of medical students and also clinicians enlisted in post-graduation and dual degree programs are also the need of the hour (38, 39).

To the best of our knowledge, this study is the first of its kind on this topic originating from King Khalid University.

This was a cross-sectional study using survey data with a small sample size; therefore, only limited generalizability of these findings can be claimed. Students from only one University were the study subjects, so future research will have to be a multi-city design to assess the extent to which it is possible to generalize the results of this study to cover all medical and dental in KSA.

## Conclusions

Based on the study's findings, undergraduate medical and dentistry students had a moderate level of positive attitudes toward conducting medical research. Lack of time, skills, funding and facilities, and limited access to medical journals and related databases were significant barriers. From this, rules must be established to identify positive behaviors and increase them in students. It is recommended to organize research workshops, training programs, frequent research presentations, and journal clubs to provide the knowledge and skills necessary for medical students to carry out scientific research in the future.

Our study discusses the challenges and barriers between medical and dentistry students to conduct medical research. Our study results and previous studies that undergraduate medical and dentistry students have a moderate level of positive attitudes toward conducting medical research, and that was due to the lack of time and skills. Funding, facilities, and limited access to medical journals and related databases are significant barriers.

Intensive training and adequate support in research activities of medical and dental students at the undergraduate level may help reduce these challenges and barriers toward research.

## Data Availability Statement

The original contributions presented in the study are included in the article/supplementary material, further inquiries can be directed to the corresponding author/s.

## Ethics Statement

The studies involving human participants were reviewed and approved by King Khalid University. The patients/participants provided their written informed consent to participate in this study.

## Author Contributions

SAAl conceived the idea of this study, supervised the study, participated in the design of the research instrument, reviewed related literature, and participated in discussing findings, making recommendations on the basis of the findings of the study, interpretation of data and writing of the manuscript. MAYA, MAAA, MIA, SSAA, YA, MAMA, and SAAs participated in data collection. MAA conceived the idea of this study, participated in the design of the study. SM finalized the manuscript for submission. All authors contributed to the article and approved the submitted version.

## Conflict of Interest

The authors declare that the research was conducted in the absence of any commercial or financial relationships that could be construed as a potential conflict of interest. The reviewer AM declared a shared affiliation, though no other collaboration, with several of the authors to the handling editor.

## Publisher's Note

All claims expressed in this article are solely those of the authors and do not necessarily represent those of their affiliated organizations, or those of the publisher, the editors and the reviewers. Any product that may be evaluated in this article, or claim that may be made by its manufacturer, is not guaranteed or endorsed by the publisher.
